# WP1130 reveals USP24 as a novel target in T-cell acute lymphoblastic leukemia

**DOI:** 10.1186/s12935-019-0773-6

**Published:** 2019-03-13

**Authors:** Hao Luo, Bo Jing, Yu Xia, Yugen Zhang, Meng Hu, Haiyan Cai, Yin Tong, Li Zhou, Li Yang, Junmei Yang, Hu Lei, Hanzhang Xu, Chuanxu Liu, Yingli Wu

**Affiliations:** 1 Hongqiao International Institute of Medicine, Shanghai Tongren Hospital/Faculty of Basic Medicine, Chemical Biology Division of Shanghai Universities E-Institutes, Key Laboratory of Cell Differentiation and Apoptosis of the Chinese Ministry of Education, Shanghai Jiao Tong University School of Medicine, Shanghai, 200025 China; 2 Department of Hematology, Shanghai First People’s Hospital, Shanghai Jiao Tong University School of Medicine, Shanghai, 200025 China; 3 State Key Laboratory of Medical Genomics, Department of Hematology, Faculty of Medical Laboratory Science, Ruijin Hospital, Shanghai Jiaotong University School of Medicine, Shanghai, 200025 China; 4 Department of Clinical Laboratory, Children’s Hospital Affiliated to Zhengzhou University, Zhengzhou, 450018 China; 5 Department of Hematology, Xinhua Hospital, Shanghai Jiao Tong University School of Medicine, Shanghai, 200092 China

**Keywords:** WP1130, USP24, dCas9-SAM, T-cell acute lymphoblastic leukemia, Mcl-1

## Abstract

**Background:**

T-cell acute lymphoblastic leukemia (T-ALL) is a lymphoid malignancy caused by the oncogenic transformation of immature T-cell progenitors with poor outcomes. WP1130 has shown potent activity against a variety of cancer but whether WP1130 has anti-T-ALL activity is not clear. USP24, one target of WP1130, is one of the largest deubiquitinases and its detailed mechanism is poorly understood. The aim of this study was to explore whether WP1130 could suppress T-ALL and the role of USP24 in T-ALL.

**Methods:**

Molecular docking and cellular thermal shift assay were performed to determine whether and how WP1130 directly interact with USP24. Mitochondrial transmembrane potential assay was measured via Rhodamine 123 staining. USP24 was reactivated using the deactivated CRISPR-associated protein 9 (dCas9)-synergistic activation mediator (SAM) system. The in vivo results were examined by tumor xenografts in NOD-SCID mice. All statistical analyses were performed with the SPSS software package.

**Results:**

WP1130 treatment decreased the viability and induces apoptosis of T-ALL cells both in vitro and in vivo. Furthermore, we demonstrated that knockdown of USP24 but not USP9X could significantly induce growth inhibition and apoptosis of T-ALL cells. Oncomine database showed that USP24 expression was upregulated in T-ALL samples and Kaplan–Meier results indicated that the USP24 was negatively but USP9X was positively associated with survival in T-ALL patients. Additionally, we proposed that WP1130 directly interacts with the activity site pocket of USP24 in T-ALL cells, which leads to the decrease of its substrates Mcl-1. Mechanistically, WP1130 induces apoptosis by accelerating the collapse of mitochondrial transmembrane potential via USP24-Mcl-1 axis.

**Conclusions:**

Altogether, using WP1130 as a chemical probe, we demonstrate that USP24 but not USP9X is a novel target in T-ALL cells. Moreover, we uncovered that WP1130 induces apoptosis by accelerating the collapse of mitochondrial transmembrane potential via USP24-Mcl-1 axis. These results provide that USP24-Mcl-1 axis may represent a novel strategy in the treatment of T-ALL and WP1130 is a promising lead compound for developing anti-T-ALL drugs.

**Electronic supplementary material:**

The online version of this article (10.1186/s12935-019-0773-6) contains supplementary material, which is available to authorized users.

## Background

T-cell acute lymphoblastic leukemia (T-ALL) is a lymphoid malignancy caused by the oncogenic transformation of immature T-cell progenitors with poor outcomes. It accounts for about 15% of pediatric and 25% of adult ALL cases, and males has higher incidence than females [1]. T cell lymphoma (TCL) and T-ALL were classified together by the World Health Organization (WHO), though the clinical presentation of them is different [2]. Despite some progress have been made, the molecular mechanisms of T-ALL development and the prognostic factors of T-ALL are still not fully understood [3, 4]. Most of the T-ALL patients will die of relapse or drug resistance. Therefore, a more effective treatment is urgently required. To this end, understanding the molecular pathogenesis of this disease may not only provide novel insights into the biology of T-ALL, but also facilitate targeted therapy for the treatment of T-ALL.

WP1130, known as Degrasyn previously [5], is a reported partially selective deubiquitinase inhibitor for USP9X, USP24 and UCH37. WP1130 has shown potent activity against a variety of cancer [6], including B-cell lymphoma (BCL) [7], glioblastoma [8], non-small cell lung cancer [9], breast cancer [10] and other malignant tumors [11–13]. However, whether WP1130 has anti-T-ALL activity is not clear.

The ubiquitin-specific protease USP24 is one of the largest deubiquitinases belonging to the ubiquitin-specific protease family. It has 2620 amino acids that contain one ubiquitin C-term hydrolase (UCH) domain as the catalytic domain and the ubiquitin-associated domain (UBA) which can bind to the ubiquitin signal on substrate proteins. A few reports show that USP24 is related with Parkinson disease [14, 15]. Through removing the ubiquitin moieties from the protein, USP24 can regulate the stability, localization and interaction of its substrates such as Mcl-1 [7], p53 [16], DDB2 [17], p300 [18] and NCOA4 [19]. Accordingly, USP24 is involved in cell death control, iron metabolism, DNA damage repair, transcription regulation and tumorigenesis [20–22]. Nevertheless, the role of USP24 in cancer is far from clear.

In this study, using WP1130 as a chemical probe, we found that USP24 but not USP9X plays important role in the survival of T-ALL cells. We further demonstrated that targeting USP24-Mcl-1 axis may represent a novel strategy in the treatment of T-ALL. Developing USP24 specific inhibitor warrants further investigation.

## Materials and methods

### Antibodies and reagents

Antibodies against USP9X (sc-365353) were purchased from Santa Cruz Biotechnology (Santa Cruz, CA). Anti-USP24 (ab72241) was purchased from Abcam. Antibodies against Mcl-1 (#94296), PARP-1 (#9532), caspase 3 (#9662), cleaved caspase-3 (#9661), K48-Ub (#8081), K63-Ub (#5621) were purchased from Cell Signaling Technology (CST). Lipofectamine 2000 (11668-019) was purchased from Invitrogen. WP1130 were purchased from Targetmol Company (Manassas, VA, USA).

### Cell culture

The human T-cell lymphoma cell lines Jurkat, Molt-4, HPB and CCRF-CEM were purchased from the ATCC (Manassas, VA, USA) and cultured in RPMI 1640 (Gibco, Carlsbad, CA, USA) supplemented with 10% fetal bovine serum (FBS; Invitrogen). All cells were cultured and maintained at 37  °C in 5% CO_2_ incubator. PBMCs were isolated from the blood of T-ALL patients or healthy volunteers by Ficoll-Hypaque (Pharmacia, Piscataway, NJ, USA) density sedimentation.

### Cell viability assay

The cell viability was detected by Cell Counting Kit-8 (Dojindo, Kumamoto, Japan). Tumor cells (3 × 10^4^/well) were seeded onto 96-well plates at a total volume (containing the WP1130 at the indicated doses) of 200 µL per well for 24 h. Then, 10 µL/well CCK-8 solutions were added and incubated with the plates for another 4 h, and the absorbance was determined at 450 nm using an MRX II microplate reader (Dynex, Chantilly, VA, USA).

### Hoechst 33342 and PI double staining

The cell death induction of WP1130 was measured using Hoechst 33342 and PI double staining. The treated cells were washed with PBS and stained with PI (10 μg/mL) and Hoechst 33342 (5 μg/mL) at room temperature for 10 min. Then the indicated cells were observed under fluorescence microscope.

### Real-time quantitative PCR

Total RNA from tissue samples was extracted using the TRIzol Kit (Invitrogen, USA) and complementary DNA was synthesized using the RT Kit (TransGen Biotech, China). RT-PCR was performed using Power SYBR Green PCR master mix (Roche, Switzerland) to detected related gene expression. Primers (Table 1) used were synthesised by Sangon Biotech (Shanghai, China). Actin was used as endogenous control to normalize the differences of total RNA in each sample.

**Table 1:** The primers sequences for genes used in the study

Name	Sequence (5′–3′)
USP9X	
F	AGGTGGTGGAATGCTTAT
R	GAGGTCTGGTGGTGATAG
USP24	
F	TGAGATGCCAGTTATTAGA
R	AGTTATCCAGCCAAGTAA
Actin	
F	CATCCTCACCCTGAAGTACC
R	AGCCTGGATAGCAACGTACAT

### Cell apoptosis assay

After cancer cells were exposed to WP1130, cells (5 × 10^5^) were centrifuged and washed twice with PBS. Cells were resuspended in 0.5 mL of cold Annexin V binding buffer, and Annexin V-FITC and Propidium iodide were added, samples were incubated at room temperature for 15 min in the dark. Annexin V positive cells were analyzed by flow cytometry using a FACScan analyzer (Becton–Dickinson, USA).

### Homology modeling and molecular docking

The homology modeling of USP24 (1689–2039) 3D structure model used SWISS-MODEL [23] (https://www.swissmodel.expasy.org/), based on the structure of USP9X (protein data bank, PDB code: 5WCH). Covalent binding between USP24 and compound WP1130 were analyzed using software AUTODOCK4. WP1130 was attached to residue CYS1698, then, during the docking, flexible side chain method was used [24]. A water molecule was docked to the binding pocket, which was centered on the residue CYS1698. The connected complex (CYS1698-WP1130) is treated as a fully flexible side chain. The binding site was covered by preparing a 60 × 60 × 60 size of grid box with grid spacing of 0.375 Å of spacing between grid points.

### Cellular thermal shift assay (CETSA)

CETSA was performed according to the method described [25]. PBS diluted Jurkat or Molt-4 cells suspensions were freeze-thawed three times with liquid nitrogen. The soluble fraction (lysate) was separated from the cell debris by centrifugation at 20,000*g* for 20 min at 4 °C. The cell lysates were diluted with PBS and divided into two aliquots, with one aliquot treated with DMSO and the other aliquot with WP1130. After 30 min incubation at room temperature the respective lysates were divided into smaller aliquots (20 μL) and heated individually at different temperatures for 3 min (Veriti thermal cycler, Applied Biosystems/Life Technologies) followed by cooling for 3 min at room temperature. The appropriate temperatures were determined in preliminary CETSA experiments (data not shown). The heated lysates were centrifuged at 20,000*g* for 20 min at 4 °C in order to separate the soluble fractions from precipitates. The supernatants were transferred to new micro tubes and analyzed by sodium dodecyl sulfate polyacrylamide gel electrophoresis (SDS-PAGE) followed by western blot analysis. Dose effect of WP1130 on the stability of USP24 was evaluated similarly.

### Mitochondrial transmembrane potential assay

After being exposed to WP1130, cells (5 × 10^5^) were centrifuged and washed twice with PBS. Cells were suspended in 0.5 mL of cold PBS, and incubated with 10 mg/L Rhodamine 123 (Rh123) at 37 °C for 30 min. Rh123 is a cationic lipophilic fluorochrome that is taken up by mitochondria in proportion to the ΔΨm. Then, 50 mg/L PI, a membrane-impermeable DNA-binding dye, was added to the cells. The fluorescent intensities were determined with flow cytometry (Becton–Dickinson). Ten thousand cells were analyzed in every sample. All data were collected, stored, and analyzed using LYSIS II software (Becton–Dickinson).

### Deactivated CRISPR-associated protein 9 (dCas9)-synergistic activation mediator (SAM) system

The cells were transfected with plenti-CMV-dspCas9-VP64 lentivirus firstly, then used puro to select after 72 h. After selected, cells successfully expressed dCas9 were then transfected with plenti-U6-sgRNA (NC or USP24) lentivirus and used blasticidin to select the positive cells after 72 h. The sequences of sgNC or sgUSP24 used in this system were list in Table 2.

**Table 2:** Sequences of dCas-sgNC or dCas-sgUSP24

Name	Sequence (5′–3′)
sgRNA^NC^	5′-GCACTACCAGAGCTAACTCA-3′
sgRNA^USP24^	5′-GAGGCGACCGTGCTCGCCGT-3′

### RNA interference and transfection

Pairs of complementary oligonucleotides (Table 3) against USP9x or USP24 and non-target control shRNA (NC) was synthesized by Sangon Biotech (Shanghai, China), annealed and ligated to the PGIPZ vector (Clontech Laboratories, Inc., CA, USA). The shRNA-carrying lentivirus which were produced in 293T cells were used to infect Jurkat cells.

**Table 3:** The sequences of shRNA targeting USP9X and USP24

Name	Sequence (5′–3′)
USP9X^NC^	5′-TTCTCCGAACGTGTCACGT-3′
USP9X^sh1^	5′-GATGTATTCTCAATCGTAT-3′
USP9X^sh2^	5′-GCCTGATTCTTCCAATGAA-3′
USP24^NC^	5′-TTCTCCGAACGTGTCACGT-3′
USP24^sh1^	5′-TGACAGTGAATAAAGATCA-3′
USP24^sh2^	5′-CCACTACTATTCCTTCATT-3′

### Western blot analysis

Cells were centrifuged and washed twice with PBS and then suspended with cold PBS, followed by addition of an equal volume of 2× cell lysis buffer. The protein concentration was quantified using the Bradford Protein Assay Kit (Thermo, Rockford, IL, USA). Cell lysates were separated by SDS-PAGE and proteins were transferred to nitrocellulose filter membranes (NC) (Millipore, Billerica, MA, USA). The membranes were then incubated with corresponding antibodies (Santa Cruz, USA) at 4  °C overnight. The membranes were washed three times with TBS/T and then incubated with the appropriate HRP-conjugated secondary antibodies for 1 h at room temperature. Protein expression was detected by chemiluminescence (GE Healthcare, Piscataway, NJ, USA).

### Xenograft mouse model

NOD-SCID mice bearing Jurkat (2 × 10^7^ per mouse) xenograft tumors were treated with vehicle or WP1130 (25 mg/kg) every 2 days after tumor volumes reached 100 mm^3^. Tumor volume was calculated with formula mm^3^ = 0.5 *a *× *b*^2^, where *a* is the length and *b* is the width. All animals were handled according to the protocols approved by the Committee for the Humane Treatment of Animals at Shanghai Jiao Tong University School of Medicine.

### Statistical analysis

A Student’s unpaired two-tailed t test was used to assess the statistical significance. Values with P < 0.05 were considered statistically significant. All statistical analyses were performed with the SPSS software package (version 17.0, SPSS, Inc., Chicago, IL).

## Results

### WP1130 inhibits proliferation and reduces viability of T-ALL cells

**Fig. 1:**
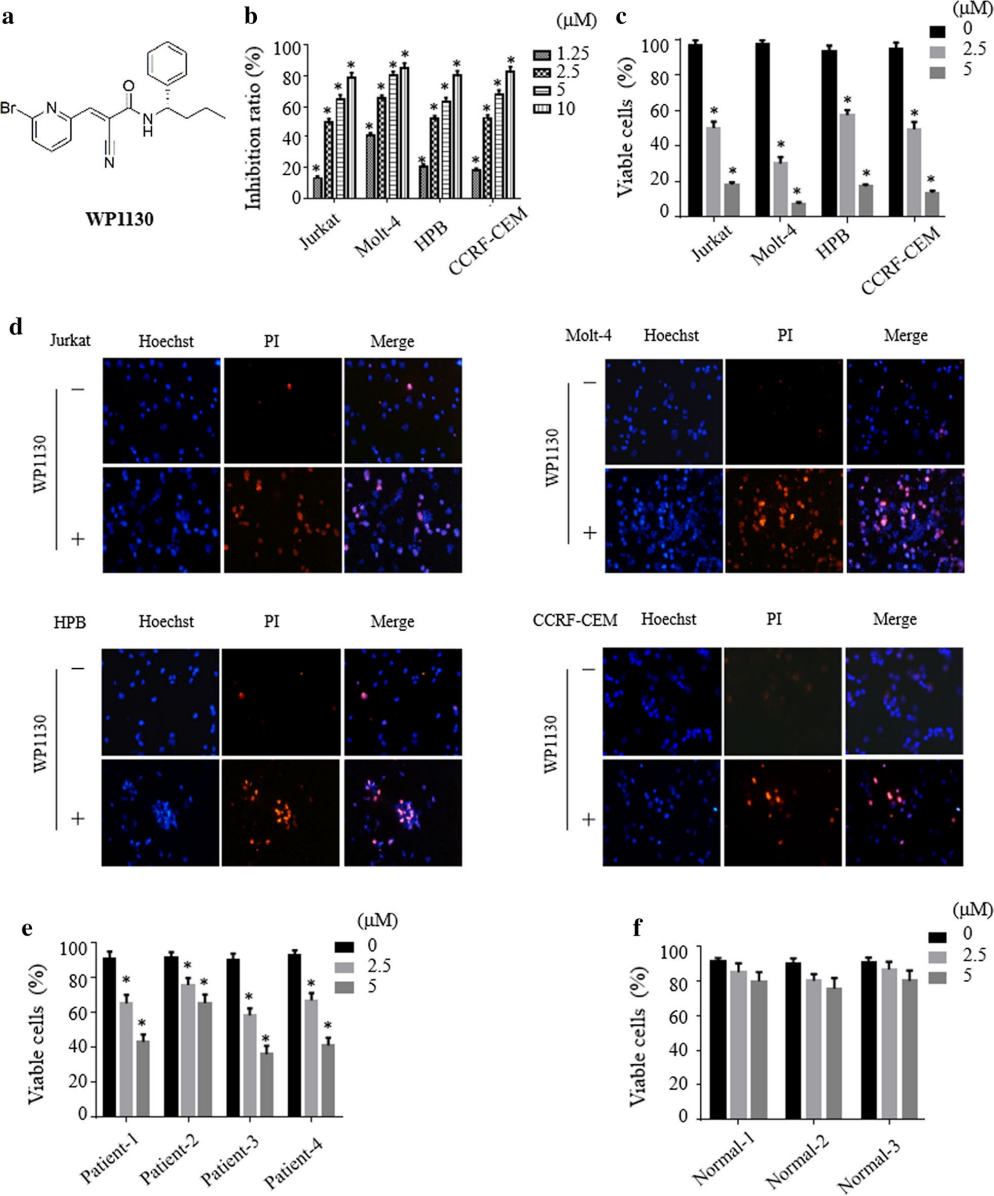
WP1130 inhibits proliferation and reduces viability of T-ALL cells. **a** Chemical structure of WP1130. **b**, **c** Jurkat, Molt-4, HPB and CCRF-CEM of different T-ALL cell lines were treated with indicated dose of WP1130 for 24 h. The inhibition of proliferation was determined by CCK-8 assays and cell viability was evaluated by trypan blue exclusion assay. **d** The treated or untreated T-ALL cell lines were stained with Hoechst 33342 and PI and then visualized by fluorescence microscope. **e**, **f** PBMCs from T-ALL patients or normal control were treated with WP1130 at indicated concentrations for 24 h and subjected to trypan blue exclusion assay to determine cell viability. All values represent mean ± SD of three independent experiments. *P < 0.05 vs. control group

To evaluate the effect of WP1130 (Fig. 1a) on the viability of T-ALL cells in vitro, Jurkat, Molt-4, HPB and CCRF-CEM were treated with various concentration of WP1130 for 24 h. Cell proliferation was determined by CCK-8 assay (Fig. 1b). Cell viability was evaluated by the trypan blue exclusion assay (Fig. 1c). As shown in Fig. 1b, c, treatment with WP1130 yielded an inhibition effect across all cell lines treated in a dose-dependent manner. We also used Hoechst-PI double staining method to detect the effect of WP1130 in T-ALL cells. Hoechst is used to stain all nuclei and PI used to stain only dead cells. As shown in Fig. 1d, WP1130 treated cells were stained with increasing PI compare to the control cells. Furthermore, treatment with WP1130 resulted in marked morphological changes (Additional file 1: Figure S1). We next evaluated the effect of WP1130 on primary T-ALL patients and PBMCs from three healthy volunteers were used as control. As shown in Fig. 1e, f, WP1130 treatment decreased the cell viability of primary monocytes from patients with T-ALL, whereas only slightly inhibited the viability in normal monocytes from healthy volunteers.

### WP1130 induces apoptosis of T-ALL cells

**Fig. 2:**
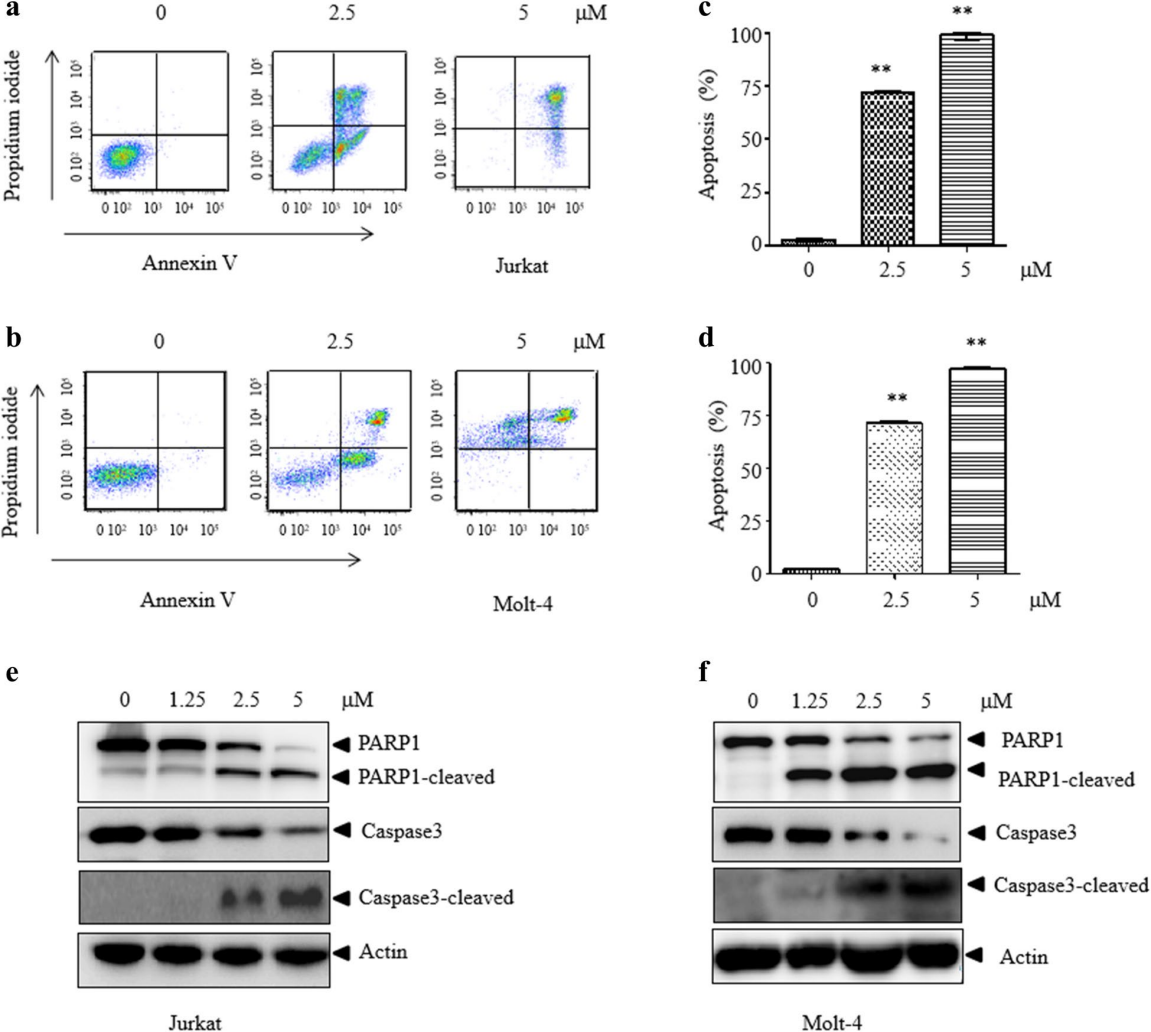
WP1130 induces apoptosis in T-ALL cells. **a**, **b** Jurkat or Molt-4 cells were exposed to WP1130 for different concentrations prior to staining with Annexin-V/PI. Apoptotic cells were assessed by flow cytometry. **c**, **d** Quantitative representation of the fraction of Annexin V-positive cells treated as described for **a** and **b** were reported. **e**, **f** Jurkat or Molt-4 cells were exposed to WP1130 and the expression of apoptosis-related protein caspase 3 and PARP1 were examined by western blot. Actin expression was determined to confirm equal protein loading. Similar results were obtained for each experiment was repeated three times. **P < 0.01 vs. control group

To determine whether WP1130 induces apoptosis in T-ALL cells, WP1130-treated cells were examined by Annexin V/PI staining. A significant increase of Annexin V positive cells was observed in Jurkat (Fig. 2a–c) and Molt-4 cells (Fig. 2b–d), indicating apoptosis induction. Consistent with this, WP1130 treatment induced cleavage of PARP1 and caspase-3, reflecting activation of apoptosis both in Jurkat (Fig. 2e) and Molt-4 (Fig. 2f) cells. These data indicate that WP1130 induces apoptosis in T-ALL cells.

### Down-regulation of USP24 but not USP9X induces growth inhibition and apoptosis of T-ALL cells

**Fig. 3:**
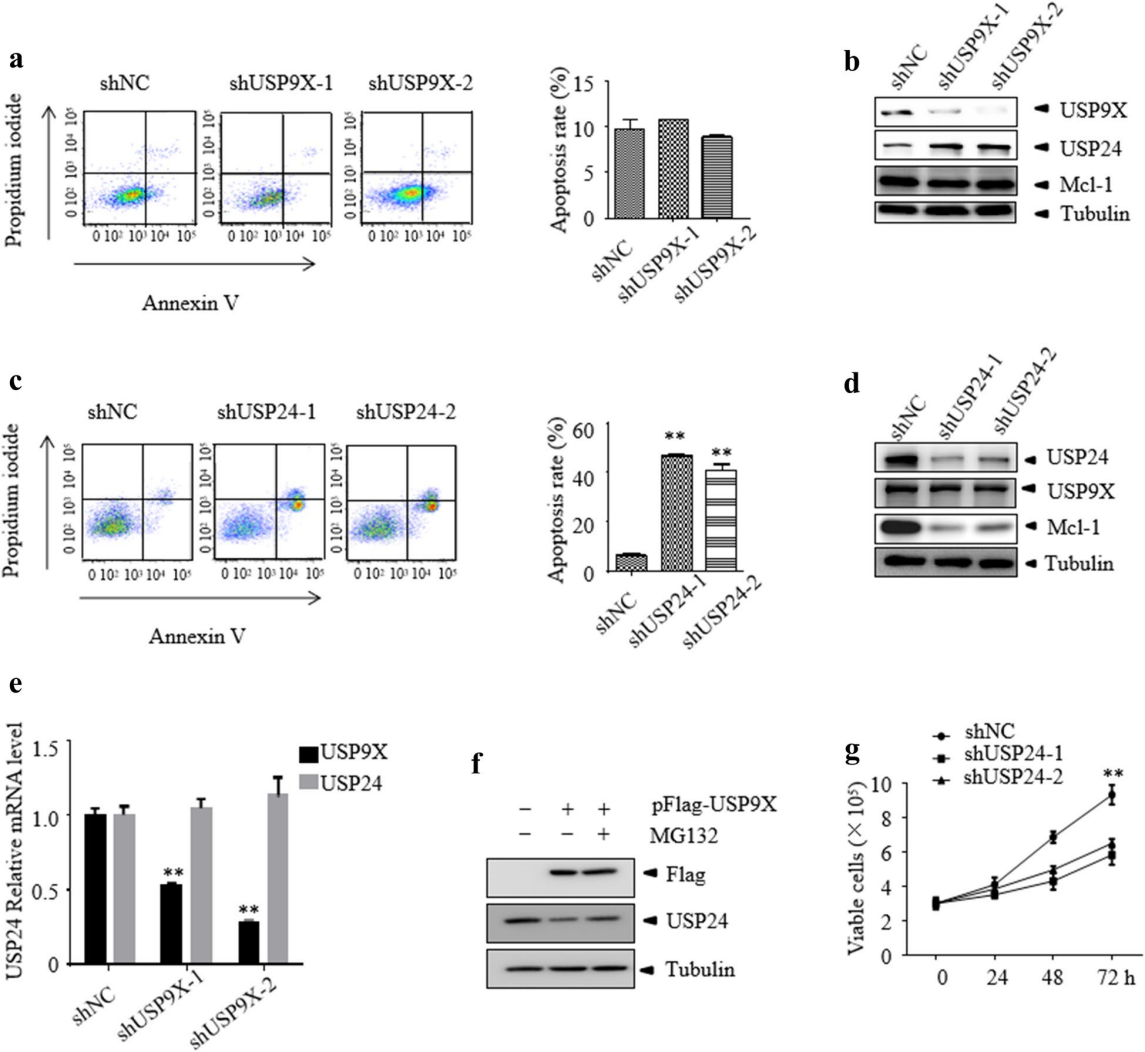
Knockdown of USP24 but not USP9X induces apoptosis and growth inhibition of T-ALL cells. **a**, **b** Jurkat cells were transfected with control or specific USP9X siRNA lentiviral for 72 h followed by flow cytometry using Annexin V and western blot assays for indicated antibodies. **c**, **d** Jurkat cells were transfected with control or specific USP24 siRNA lentiviral for 72 h followed by flow cytometry using Annexin V and western blot assays for indicated antibodies. **e** The mRNA level of USP9X and USP24 was assessed by RT-PCR. **f** USP9X was over-expressed in 293T and treated in the presence or absence of MG132, then the indicated protein were determined by western blotting. **g** Cell viability was monitored by trypan blue staining. All the date shown is representative of values from at least three independent experiments with similar result. **P < 0.01 vs. control group

WP1130 was reported to act as an inhibitor of USP9X, USP24 and some other DUBs [7, 26]. To further evaluate the role of USP9X in WP1130-induced cell death of T-ALL, two USP9X specific shRNA and the control non-specific shRNA were stably transfected into Jurkat cells. The knockdown efficacy was determined by western blot and cell survival was examined by Annexin V/PI staining (Fig. 3a). USP9X protein was significantly reduced in Jurkat cells. However, knockdown of USP9X did not change cell viability and the anti-apoptotic protein Mcl-1 remains unchanged, which was reported as a substrate of USP9X [27] (Fig. 3b). Previous report showed that the expression level of Mcl-1 was regulated by USP24 in USP9X knockdown B-cell malignancies [28]. Therefore, we probed for the expression level of USP24 in USP9X silenced Jurkat cells. As expected, USP24 was upregulated in USP9X silenced cells (Fig. 3b) and we also found this upregulation did not occur at the mRNA level (Fig. 3e). In order to determine whether USP9X promote the degradation of USP24, we over-expressed USP9X in 293T cells and examined the protein level of USP24. As shown in Fig. 3f, when USP9X was over-expressed, the protein level of USP24 decreased. Moreover, the decreasing of USP24 could be rescued by MG132, indicating that USP9X can promote the degradation of USP24. Interestingly, knockdown of USP24 in Jurkat cells significantly induced cell death (Fig. 3c) and reduced Mcl-1 but not USP9X protein level (Fig. 3d). Furthermore, knockdown of USP24 markedly inhibits the proliferation of Jurkat cells (Fig. 3f). These data suggest that reduced USP24 may contribute to WP1130-induced cell death of T-ALL cells and USP24 may serve as a novel target to the treatment of T-ALL.

### USP24 is overexpressed in T-ALL and negatively associated with survival in patients with T-ALL

**Fig. 4:**
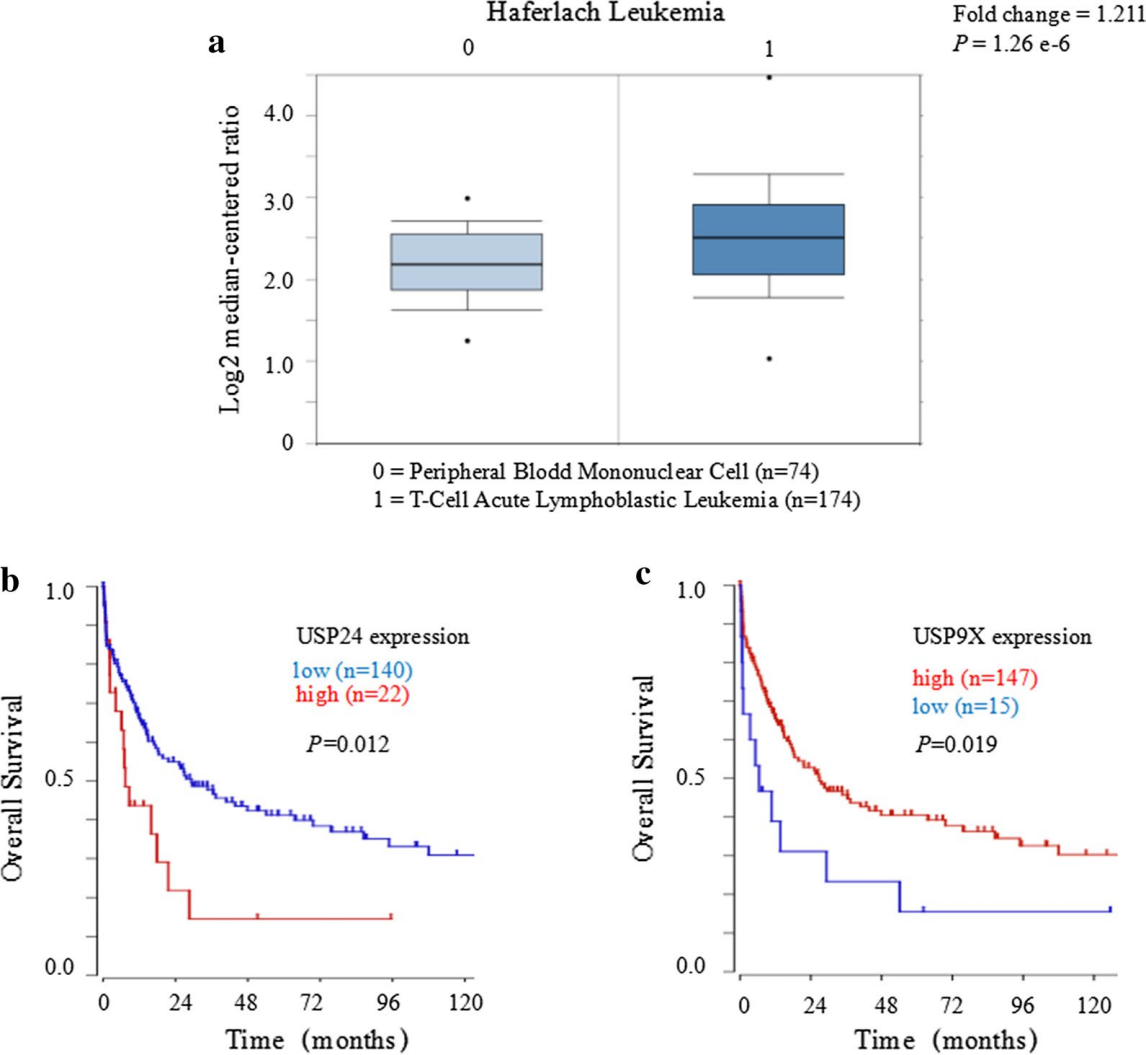
USP24 is upregulated and has negative correlation with the overall survival in T-ALL/TCL patients. **a** Analysis of TCGA leukemia dataset from the Oncomine database to assess the expression of USP24 in normal peripheral blood cells and in T-ALL patient samples. The data are presented with box plots. Fold change, P-value (determined by Student’s t-test), and sample size are shown. **b**, **c** The prognostic value of USP24 and USP9X expression at the mRNA level was verified according to the data of R2 microarray database (https://hgserver1.amc.nl/cgi-bin/r2/main.cgi), and this results indicated that the lower expression of USP24 but higher expression of USP9X at the mRNA level was significantly associated with a better survival of the T-ALL/TCL patients (P < 0.05)

To extend the above observation, using TCGA leukemia datasets from the public Oncomine database (https://www.oncomine.org/) we carried out bioinformatics analysis. As shown in Fig. 4a, USP24 was upregulated in T-ALL samples compared with its expression in normal peripheral blood cells. WHO classified TCL and T-ALL together though the different clinical presentation [2]. Despite relevant data on the prognosis of USP24 and USP9X in T-ALL are unavailable currently, the big data analysis using R2 database (http://hgserver1.amc.nl) affect the prognosis of TCL patients shows that the mRNA gene expression level of USP24 was negatively but USP9X was positively associated with survival in patients with TCL (Fig. 4b, c). Interestingly, the Kaplan–Meier results indicated that the prognostic of USP24 and USP9X expression pattern was opposite in TCL. Taken together, these findings suggest that USP24 plays a critical function in the T-ALL cell survival and targeting USP24 but not USP9X may thus be a novel approach for the treatment of T-ALL.

### WP1130 interacts with USP24 in cells

**Fig. 5:**
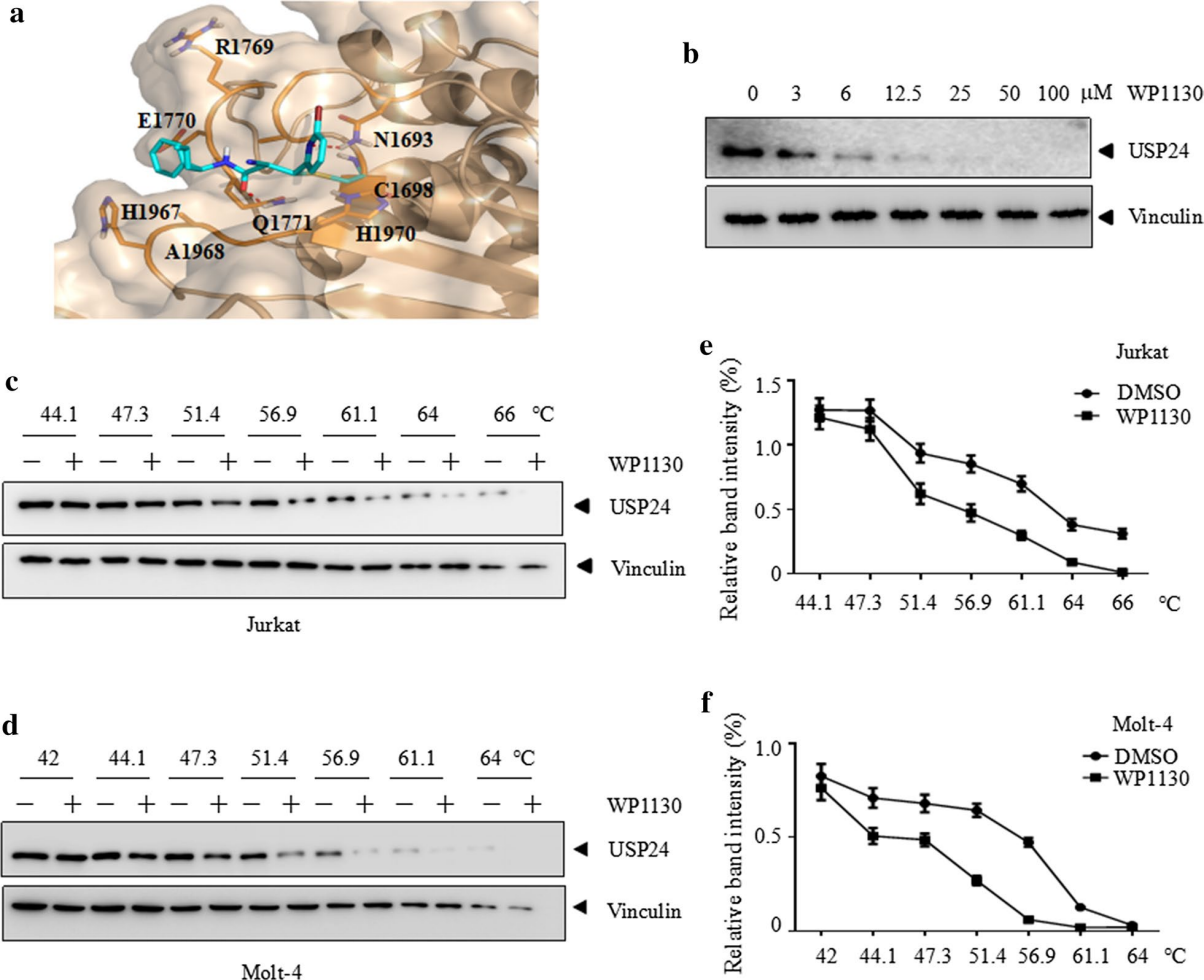
WP1130 interacts with USP24 in cells. **a** Predicted binding model of WP1130 in USP24 pocket. The dashed lines in red represent hydrogen bonds between WP1130 (cyan) and USP24 (wheat). **b**–**d** CETSA was performed on indicated cells as described in methods. The thermal stabilization effect of WP1130 on USP24 and vinculin at different doses and different temperatures were evaluated by western blot. **e**, **f** The intensity of the bands of **c** and **d** were quantified by Image J software. All experiments were repeated three times with the same results

To elucidate the interaction between WP1130 and USP24, a molecular docking study was performed. A covalent binding model of compound WP1130 was generated on the modeled structure of USP24 by AUTODOCK4. It demonstrated that the compound binds to a pocket that constituted by Asn1693, Cys1698, Arg1769, Glu1770, Gln1771, His1967, Ala1968 and His1970. WP1130 covalent bound to the residue Cys1698. Residue Asn1693 and Gln1771 form polar interactions to the small compound (Fig. 5a).

In order to investigate whether the WP1130 directly interacts with USP24, a cellular thermal shift assay (CETSA) was conducted [25, 29]. As shown in Fig. 5b, with the presence of increasing concentrations of WP1130, the thermal stability of USP24 markedly decreased. Consistent with this, WP1130 markedly decreased the thermal stability of USP24 at different temperatures in Jurkat (Fig. 5c, e) and Molt-4 (Fig. 5d, f) cells. As a negative control, WP1130 did not change the thermal stability of vinculin in cells. These data suggest that WP1130 directly interacts with USP24 in T-ALL cells.

### WP1130 induces apoptosis by accelerating the collapse of mitochondrial transmembrane potential via USP24-Mcl-1 axis

**Fig. 6:**
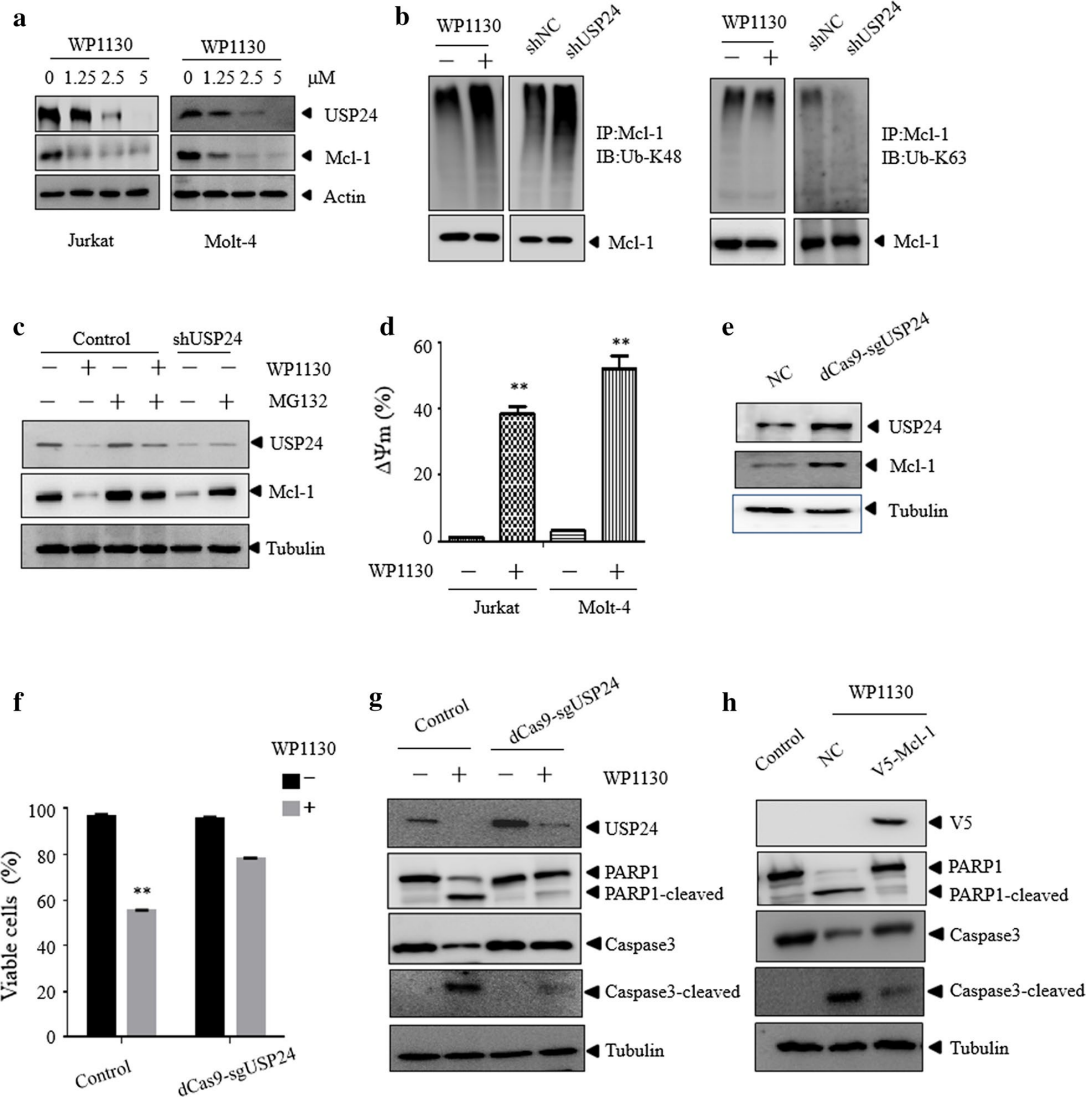
WP1130 induces apoptosis via USP24-Mcl-1 axis. **a** Different doses of WP1130 treated on the expression of indicated proteins in Jurkat and Molt-4 cells. Cell lysates were subjected to western blotting analysis. **b** Mcl-1 was immunoprecipitated from Jurkat cells in the presence and absence of WP1130 or knockdown of USP24, and the indicated proteins were examined by western blot. **c** The indicated cells were treated with WP1130 in the presence or absence of MG132, and the protein level of USP24 and Mcl-1 were then examined by western blotting. **d** Jurkat or Molt-4 cells were exposed to DMSO or WP1130 for 24 h. The collapse of mitochondrial transmembrane potential was assessed by flow cytometry using RH123 staining. **e**–**g** Jurkat cells were transfected with the vector or dCas9-sgUSP24 plasmid, followed by western blot assay. Indicated cells were treated with WP1130 and the cell viability was evaluated by trypan blue exclusion assay and the indicated protein was examined by western blot. **h** Jurkat cells were transfected with the vector or V5-Mcl-1 plasmid and then treated with WP1130 and the indicated proteins were examined by western blot. Similar results were obtained for each experiment was repeated three times. **P < 0.01 vs. control group

We further explored the mechanism by which WP1130 induced apoptosis of T-ALL cells. It has been demonstrated that USP24 could stabilize Mcl-1 protein by deubiquitinating Mcl-1 [7]. WP1130 treatment significantly reduced the protein level of USP24 and Mcl-1 in Jurkat and Molt-4 (Fig. 6a) cells. However, WP1130 did not reduce the mRNA level of USP24 (Additional file 2: Figure S2). As shown in Fig. 6b, WP1130 treatment or USP24 knockdown resulted in the increased K48-linked ubiquitination of Mcl-1. Moreover, as shown in Fig. 6c, MG132 could block the proteasome degradation of Mcl-1 when USP24 was inhibited or knocked down. As a member of Bcl-2 protein family, Mcl-1 plays an important role in maintaining mitochondrial transmembrane potential and promoting cell survival [30]. Thus, we assumed that WP1130 may induce T-ALL cells death by targeting USP24 and reducing the expression of Mcl-1 protein, which in turn resulted in the collapse of mitochondrial transmembrane potential. As expected, the mitochondrial membrane potential was significantly decreased in WP1130 treated cells (Fig. 6d). To determine whether the reduction of USP24 was associated with apoptosis of T-ALL cells, USP24 was reactivated used the Cas9-based activators method [31] in Jurkat cells (Fig. 6e). As shown in Fig. 6f, g, reactivation of USP24 markedly prevented Jurkat cell apoptosis induced by WP1130. Furthermore, overexpression of Mcl-1 significantly abrogated the apoptosis effect of WP1130 (Fig. 6h). These data indicate that WP1130 induces apoptosis by accelerating the collapse of mitochondrial transmembrane potential via USP24-Mcl-1 axis.

### WP1130 inhibits T-ALL cells growth in vivo

**Fig. 7:**
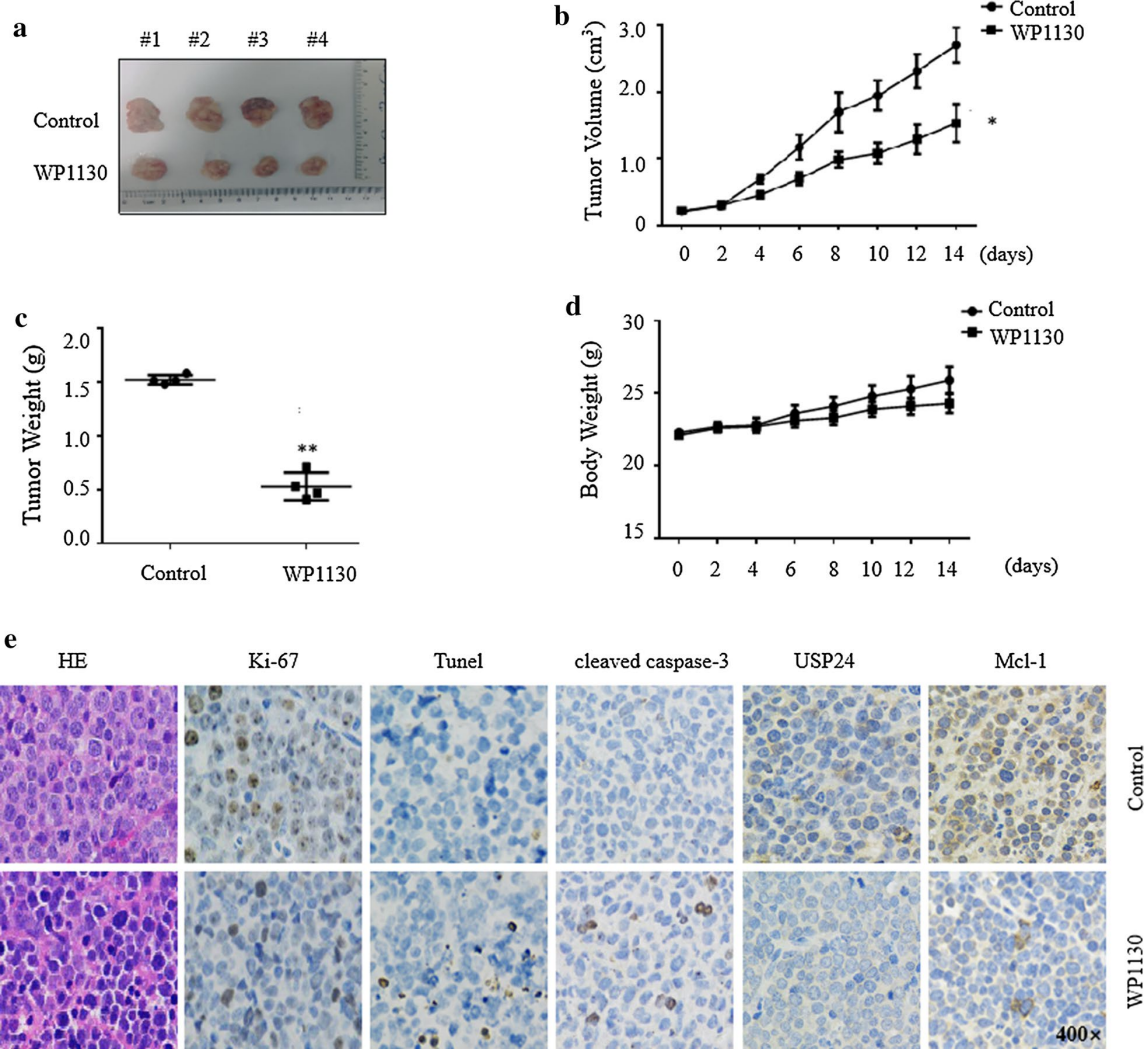
WP1130 inhibits T-ALL cells growth in vivo. **a** Image of xenograft tumors treated with WP1130 or control on day 14. **b** Tumor growth curves were recorded every 2 days in two groups. **c** Effect of WP1130 on xenograft tumor weight. **d** Effect of WP1130 on mouse body weight. Values are expressed as the mean ± SD, n = 4 for each group. **e** The expression patterns of Ki-67, Tunel, cleaved caspase-3, USP24 and Mcl-1 were tested by immunohistochemistry analysis in the xenograft tumors on day 14 in each group. Original magnification, w400. **P < 0.01 vs. control group. *P < 0.05 vs. control group

Finally, we determined the efficacy of WP1130 in vivo using a xenograft T-ALL model. Jurkat cells were subcutaneously implanted into NOD-SCID mice. When the tumor reaches 100 mm^3^, the mice were treated with WP1130 or vehicle. Compared with the control group, the intra-peritoneal injection of WP1130 significantly suppressed the tumor growth (Fig. 7a–c). WP1130 at 25 mg/kg was tolerant in mice, although a slight decrease of body weight was observed in WP1130-treated group (Fig. 7d). This effect might be due to inhibition of cell proliferation and induction of cell death, as indicated by decrease of Ki-67 staining and increase of TUNEL and cleaved caspase-3 positive cells (Fig. 7e). Consistent with in vitro assay, WP1130 treatment also lead to the decreased of USP24 and Mcl-1 levels in Jurkat tumor xenografts (Fig. 7e). Collectively, these results demonstrate that WP1130 could inhibit T-ALL cell growth and USP24 activity in vivo.

## Discussion

**Fig. 8:**
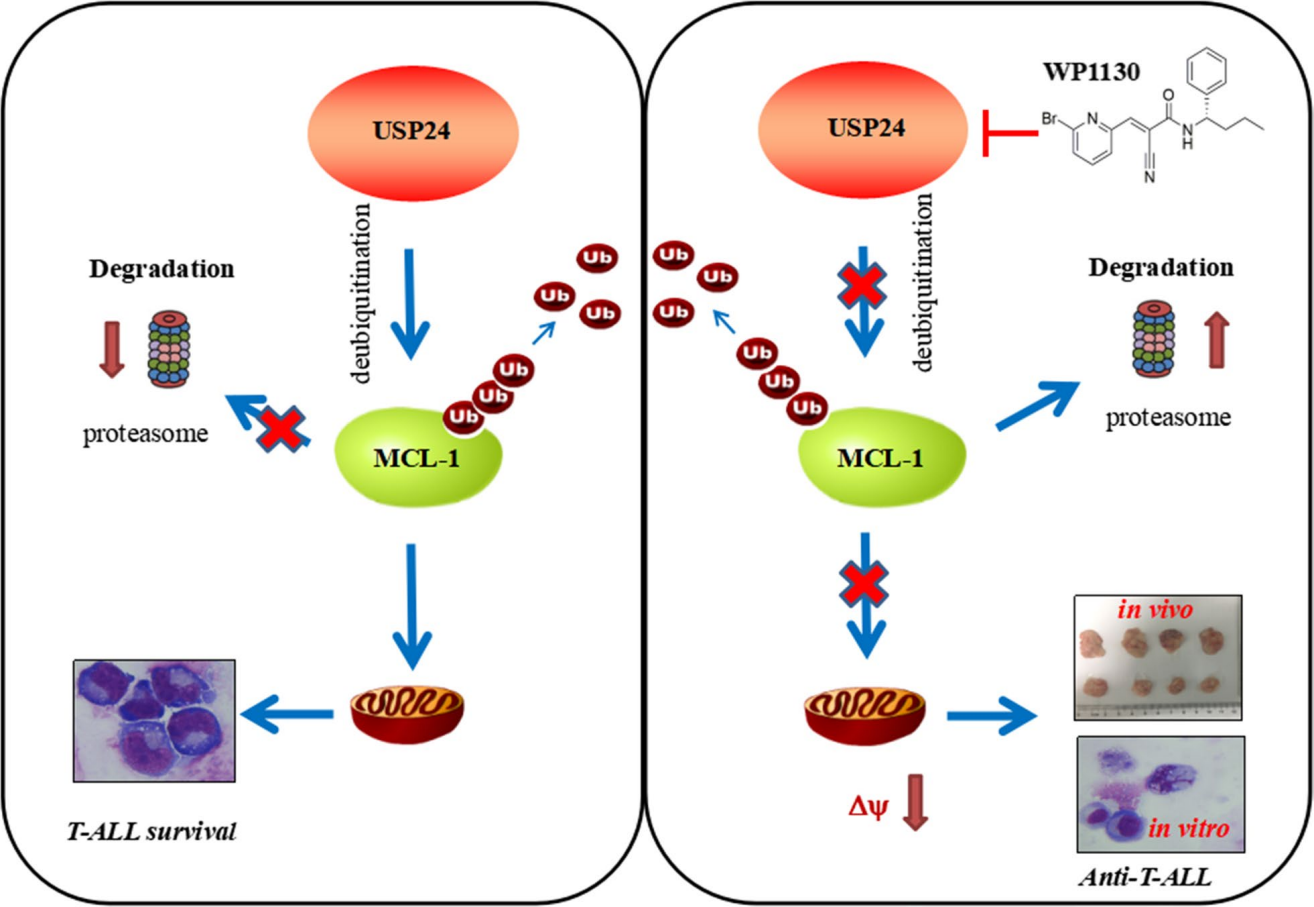
Schematic representation of the mechanisms underlying apoptosis induced by WP1130 targeting USP24 in T-ALL. USP24 play an important role in the survival of T-ALL by deubiquitinating of its substrate Mcl-1 which helps to maintain mitochondrial integrity. WP1130 directly interacts and inhibits the deubiquitination activity of USP24, which in turn increasing the degradation of the it’s substrate Mcl-1, and ultimately accelerating the collapse of mitochondrial transmembrane potential that induce apoptosis of T-ALL cells both in vivo and in vitro

T-ALL is a neoplasm derived from T cells. Owing to severe immunosuppression and intrinsic chemoresistance patients with this disease have poor overall survival. Despite some advances such as combination of chemotherapy, complete remission and overall survival remains no significant improvement. Therefore, understanding the molecular pathogenesis deeply and facilitating more effective treatment is urgently required for this disease. In our study, we found that WP1130 could effectively inhibit the survival T-ALL cells both in vitro and in vivo and revealed USP24 but not USP9X as a novel target for T-ALL treatment (Fig. 8).

Notably, WP1130 is not only inhibiting USP9X, but also other DUBs such as USP24, an USP9X closely related DUB [7]. Role of USP9X in cancer is tissue specific and USP9X displays both oncogenic and tumor suppressor activities [32–34]. Interestingly, different from Peterson’s finding that USP9X is an oncogene in B-cell malignancies [7], we found that knockdown of USP9X did not induce cell death of T-ALL cells, indicating that the role of USP9X is cell type dependent. Consistent with our findings, Spinella et al. reported that USP9X may play a tumor suppressor role in T-ALL [35]. It is interesting to observe that knockdown of USP9X resulted in a feedback upregulation of USP24, which may keep cell alive through maintaining the stability of Mcl-1. Interestingly, we found that knock down of USP9X shows no effect but knock down of USP24 could recapitulates the effects of WP1130 in T-ALL cells. Currently, the mechanism of knockdown of USP9X-induced upregulation USP24 is not known. However, we showed that the upregulation of USP24 does not occur at transcription level, as knockdown of USP9X does not change the mRNA level of USP24. Our preliminary data showed that USP9X overexpression resulted in the degradation of USP24, suggesting that USP9X may regulate the stability of USP24.

The function of USP24 in cancer is poorly understood. Accordingly, USP24 could function as a tumor suppressor or oncogene in different kind of cancers or at different stage of cancer progression [18, 20]. A previous study reported that USP24 can stabilize MDM2 then decrease the Suv39h1 level resulting in lung cancer metastasis [21]. We showed that USP24 play an important role in the survival of T-ALL cells. Using Oncomine database to assess the expression of USP24 in T-ALL, we found its expression was upregulated compare to normal. T cell lymphoma (TCL) and T-ALL are considered the same disease, differing by the extent of bone marrow infiltration [2]. We performed an analysis using the R2 microarray database and found that the expression of USP24 was negatively but USP9X was positively associated with survival in patients with TCL. Taken together, these findings suggest that the functional role of USP9X/USP24 is cell type dependent and USP24 play a critical role in the T-ALL cell survival. Considering the possible tumor suppressor role of USP9X and oncogene role of USP24 in T-ALL and the feedback regulation between USP9X and USP24, developing a USP24 specific inhibitor is needed.

In our study, using CETSA we have shown that WP1130 can direct target USP24 in T-ALL cells. Performing molecular docking assessments, we predicted that WP1130 covalently bound to residue Cys1698, and formed polar interactions with residues Asn1693 and Gln1771. Of note, a better understanding of binding mode may be achieved through the crystal structure in future pursuits. Consistent with the inhibition of USP24 activity, treatment of T-ALL cells with WP1130 decreased the levels of its substrates Mcl-1 [7].Therefore, development of a new selective USP24 inhibitor may be a promising therapeutic strategy for T-ALL.

A robust and rapid reactivation system, which used catalytically-deficient Cas9 (dCas9)-synergistic activation mediator (SAM), was been widely used recently [36–38]. Using this deactivated CRISP-Cas9-SAM system we successfully reactivated USP24 and demonstrate that endogenously induced USP24 could partially rescue the apoptosis induced by WP1130 in Jurkat cells. Furthermore, we show that the mitochondrial apoptotic pathway is involved in WP1130 induced cell death in T-ALL cells. This is most likely due to the downregulation of Mcl-1 protein. Mcl-1 belongs to the anti-apoptotic proteins of the Bcl-2 family [39]. It helps to maintain mitochondrial integrity, prevent the release of pro-apoptotic factors from the intermembrane space into the cytosol and the subsequent caspase activation [40]. Therefore, our data suggests that WP1130 induces apoptosis by accelerating the collapse of mitochondrial transmembrane potential via USP24-Mcl-1 axis.

## Conclusion

Taken together, using WP1130 as a chemical probe, we demonstrate that USP24 but not USP9X is a novel target in T-ALL cells. Moreover, we uncovered that WP1130 induces apoptosis by accelerating the collapse of mitochondrial transmembrane potential via USP24-Mcl-1 axis. These results provide evidence that targeting USP24-Mcl-1 axis may represent a novel strategy in the treatment of T-ALL and WP1130 is a promising lead compound for developing anti-T-ALL drugs.

## Additional files

**Additional file 1: Figure S1.** The representative morphology of T-ALL cells treated with WP1130 was monitored by Wright’s staining.

**Additional file 2: Figure S2.** Jurkat or Molt-4 cells were treated with WP1130 for 24 h, and the mRNA of USP24 was examined by RT-PCR.

## Abbreviations

T-ALL: T-cell acute lymphoblastic leukemia
TCL: T cell lymphoma
Rh123: Rhodamine 123
PI: propidium iodide
dCas9: deactivated CRISP associated protein 9
SAM: synergistic activation mediator
CETSA: cellular thermal shift assay
UCH: ubiquitin C-term hydrolase
UBA: ubiquitin-associated domain
