# Supplementary figures and images for: WP1130 reveals USP24 as a novel target in T-cell acute lymphoblastic leukemia
Source: Cancer Cell Int. 2019 Mar 13;19:56. doi: 10.1186/s12935-019-0773-6 (PMC6415346; doi:10.1186/s12935-019-0773-6)

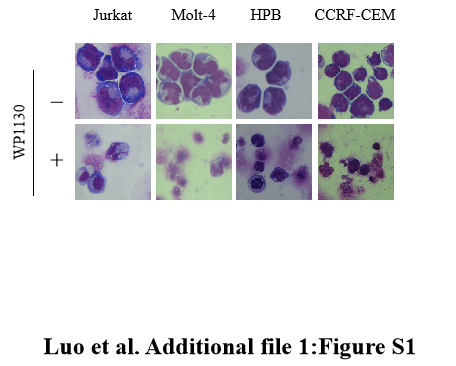

Supplement: Supplementary file 1 — Additional file 1: Figure S1. The representative morphology of T-ALL cells treated with WP1130 was monitored by Wright’s staining. [file 12935_2019_773_MOESM1_ESM.tif]

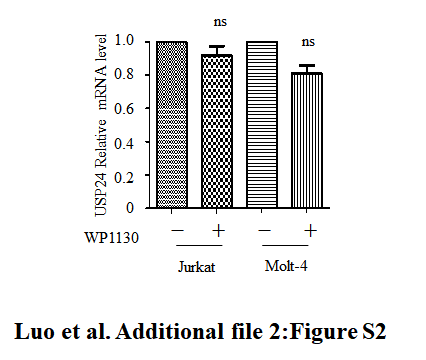

Supplement: Supplementary file 2 — Additional file 2: Figure S2. Jurkat or Molt-4 cells were treated with WP1130 for 24 h, and the mRNA of USP24 was examined by RT-PCR. [file 12935_2019_773_MOESM2_ESM.tif]
